# Dexmedetomidine inhibits astrocyte pyroptosis and subsequently protects the brain in in vitro and in vivo models of sepsis

**DOI:** 10.1038/s41419-019-1416-5

**Published:** 2019-02-18

**Authors:** Yi-Bing Sun, Hailin Zhao, Dong-Liang Mu, Wenwen Zhang, Jiang Cui, Lingzhi Wu, Azeem Alam, Dong-Xin Wang, Daqing Ma

**Affiliations:** 1 Department of Anesthesiology and Critical Care Medicine, Peking University First Hospital, Beijing, China; 2 Anaesthetics, Pain Medicine and Intensive Care, Department of Surgery and Cancer, Faculty of Medicine, Imperial College London, Chelsea and Westminster Hospital, London, UK; 3 Department of Obstetrics and Gynecology, the Second Affiliated Hospital of Wenzhou Medical University, Wenzhou, Zhejiang Province China

## Abstract

Sepsis is life-threatening and often leads to acute brain damage. Dexmedetomidine, an α_2_-adrenoceptor agonist, has been reported to possess neuroprotective effects against various brain injury but underlying mechanisms remain elusive. In this study, in vitro and in vivo models of sepsis were used to explore the effects of dexmedetomidine on the inflammasome activity and its associated glia pyroptosis and neuronal death. In vitro, inflammasome activation and pyroptosis were found in astrocytes following lipopolysaccharide (LPS) exposure. Dexmedetomidine significantly alleviated astrocyte pyroptosis and inhibited histone release induced by LPS. In vivo, LPS treatment in rats promoted caspase-1 immunoreactivity in astrocytes and caused an increase in the release of pro-inflammatory cytokines of IL-1β and IL-18, resulting in neuronal injury, which was attenuated by dexmedetomidine; this neuroprotective effect was abolished by α_2_-adrenoceptor antagonist atipamezole. Dexmedetomidine significantly reduced the high mortality rate caused by LPS challenge. Our data demonstrated that dexmedetomidine may protect glia cells via reducing pyroptosis and subsequently protect neurons, all of which may preserve brain function and ultimately improve the outcome in sepsis.

## Introduction

Sepsis, a severe systemic inflammatory response as a result of infection, is associated with high morbidity and mortality rates. It is the most common reason for admission to an intensive care unit and is one of the leading causes of death. Sepsis-induced brain injury occurs in the early stage of sepsis in critically ill patients, and contributes to the progression of sepsis^1^. Patients with sepsis-induced brain injury often have changes in mental state, ranging from delirium to coma, accompanied by altered levels of consciousness, cognition, and perceptual abnormalities. Postmortem analyses demonstrate that the pathophysiology of sepsis-induced brain injury often shows diffuse brain microabscesses, indicating that the injury is likely associated with bacterial and/or endotoxin direct invasion to the brain^2^. Growing evidence indicates that this process involves inflammatory mechanisms leading to the central nervous system (CNS) dysfunction^3,4^, neuroinflammation and related excitotoxicity are involved in the pathological process of neurological dysfunction induced by sepsis. In addition, astrocytes play a critical role in providing functional support for neurons and regulate the communication with adjacent neurons via gliotransmission, synaptic plasticity, gap junctions, and energy metabolic support^5^. Furthermore, astrocytes participate in brain injury associated with neuroinflammation through the release of pro-inflammatory cytokines and toxic molecules, whilst the activation of astrocytes results in detrimental effects on neuronal function and can cause deleterious neurological sequalae^6,7^. Therefore, the protection of astrocytes could be a potential therapeutic target against sepsis-induced brain injury.

Dexmedetomidine, an α_2_-adrenoceptor agonist, is widely used in the perioperative period for critically ill patients in the intensive care unit for sedation, analgesia, and anxiolysis. A previous study showed that patients treated with dexmedetomidine had a greater number of ventilator-free hours than the placebo-treated group^8^. It has been even suggested that the choice of dexmedetomidine rather than a benzodiazepine is strongly recommended for preventing delirium in critically ill patients^9^. Recent evidence also suggests that dexmedetomidine increases survival up to 2 years for elderly patients admitted to ICU, as well as improves cognitive function and quality of life in 3-year survivors^10^. In addition, protective effects of dexmedetomidine have been reported against apoptosis, necrosis, and autophagy in the brain and peripheral tissues in various in vitro and in vivo models^11–13^. Dexmedetomidine has been shown to provide protection against bilirubin-induced lung damage by inhibiting cell apoptosis and promoting cell survival^12^. In addition, dexmedetomidine-mediated protection against brain injury induced by ischemia-reperfusion injury has been demonstrated, which is thought to be mediated by autophagy suppression^13^. However, whether dexmedetomidine can inhibit pyroptosis in the central nervous system in sepsis setting remains unknown.

Pyroptosis is a pro-inflammatory form of cell death and a regulated form of necrosis, distinct from other forms of cell death associated with inflammasome activation. The inflammasome functions as a sensor to detect invading pathogens and cellular damage danger signals, and induces the excessive production of potent pro-inflammatory cytokines, which augments inflammation-mediated organ injury^14^. Cells undergoing pyroptosis experience cellular swelling, rapid membrane rupture, DNA fragmentation, and extravasation of their intracellular contents, resulting in toxicity to adjacent healthy cells and causing further cell death. Pyroptosis can be triggered by various pathological stimuli, such as microbial infection and ischemia reperfusion^15,16^. Accumulating evidence has demonstrated that this novel type of cell death may play a critical role in the pathogenesis of central nervous system diseases, including Alzheimer’s disease and multiple sclerosis, which are characterized by neuronal damage and neuroinflammation^17^. Furthermore, it has been found that a range of cell types in the CNS may undergo inflammasome-induced pyroptosis^18,19^. These cells, including microglia and astrocytes, exhibit NLRP3 inflammasome-related responses, which may be responsible for the deterioration in neurologic function^18,19^. Studies have demonstrated that dexmedetomidine may alleviate the inflammatory response by reducing inflammasome activation in the liver and pancreas^20,21^; however, the anti-inflammatory effects of dexmedetomidine associated with pyroptosis in sepsis-induced brain injury require further investigation. In this study, we set out to evaluate the hypothesis that dexmedetomidine may protect the brain via inhibiting glia pyroptosis in in vitro and in vivo models of sepsis.

## Results

### LPS but not TNF-α induces NLRP3 inflammasome activation and pyroptosis in cultured astrocytes

**Fig. 1:**
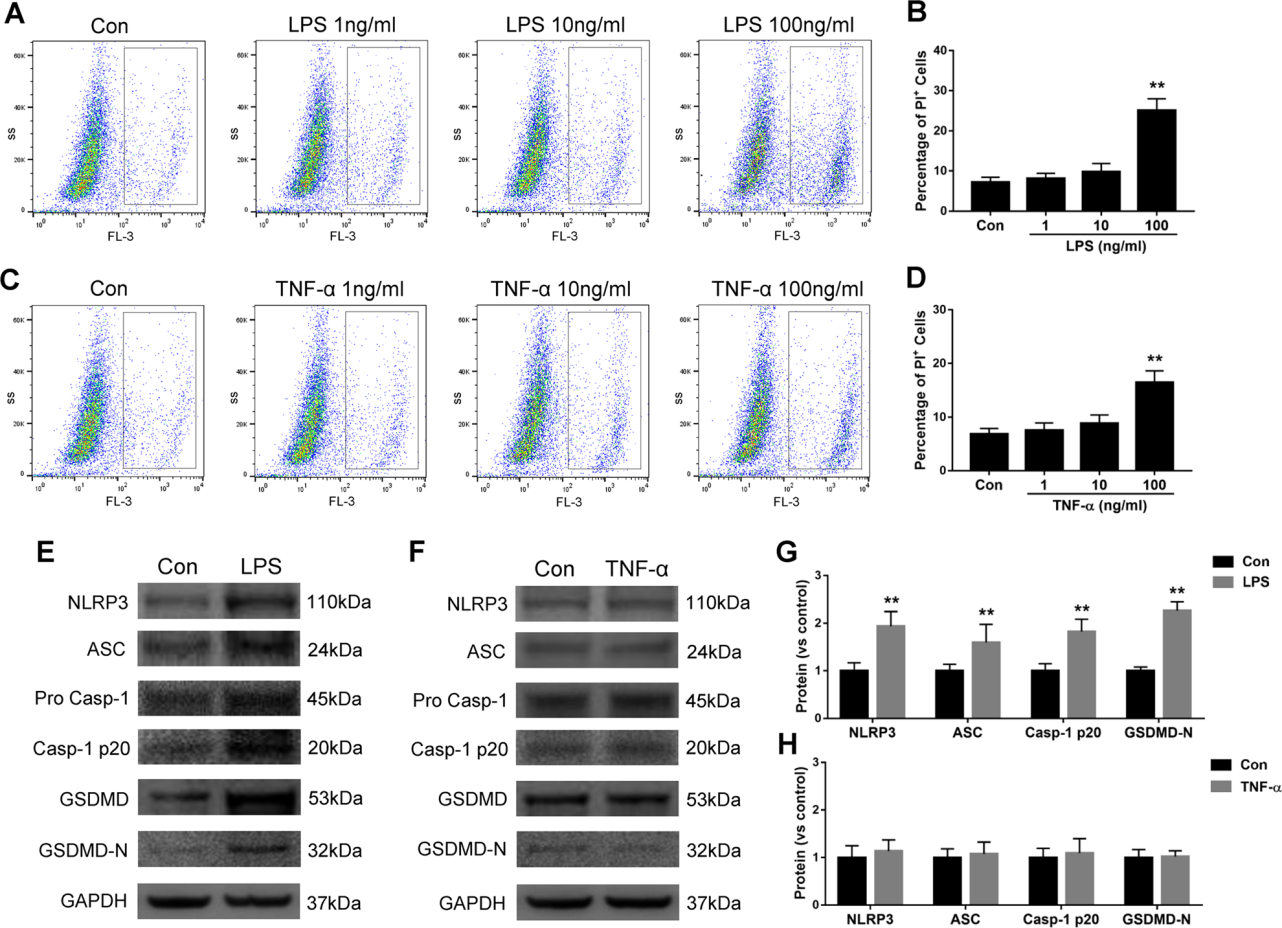
NLRP3 inflammasome activation and pyroptosis induced by LPS, but not TNF-α, in cultured astrocytes. 1321N1 cells were treated with LPS (1–100 ng/ml) **a**, **b** or TNF-α (1–100 ng/ml) **c**, **d** for 24 h. The percentage of 1321N1 dead cells was measured with propidium iodide (PI) staining followed by flow cytometry analysis. Western blotting analysis of NLRP3, ASC, pro-caspase-1, caspase-1-p20, GSDMD, GSDMD-N in the presence of LPS **e**, **g** or TNF-α **f**, **h**. Data are represented as mean ± SD (*n* = 6). **p < 0.01, vs. the control group

To explore the cytotoxicity of pathogenic mediators of sepsis, the 1321N1 astrocytes were cultured with LPS or TNF-α as an extrinsic and intrinsic mediator, respectively, followed by propidium iodide (PI) staining and flow cytometry assessment. Both LPS and TNF-α caused cell death in a dose-dependent manner (*p* < 0.01) (Fig. 1a–d). Further, the pyroptosis-related proteins were assessed. As shown in the western blot analysis, LPS treatment stimulated the upregulation of NLRP3 (nucleotide-binding domain, leucine-rich repeat containing protein 3), ASC (apoptosis-associated speck-like protein containing a CARD), caspase-1 and GSDMD (gasdermin D) expression (*p* < 0.01, Fig. 1e, g). Those proteins were not increased in the TNF-α cultured astrocytes (*p* > 0.05, Fig. 1f, h), indicating that caspase-1 activation and pyroptosis occurred with LPS, but not TNF-induced astrocyte cell death.

### Dexmedetomidine attenuates LPS-induced pyroptosis in cultured astrocytes

**Fig. 2:**
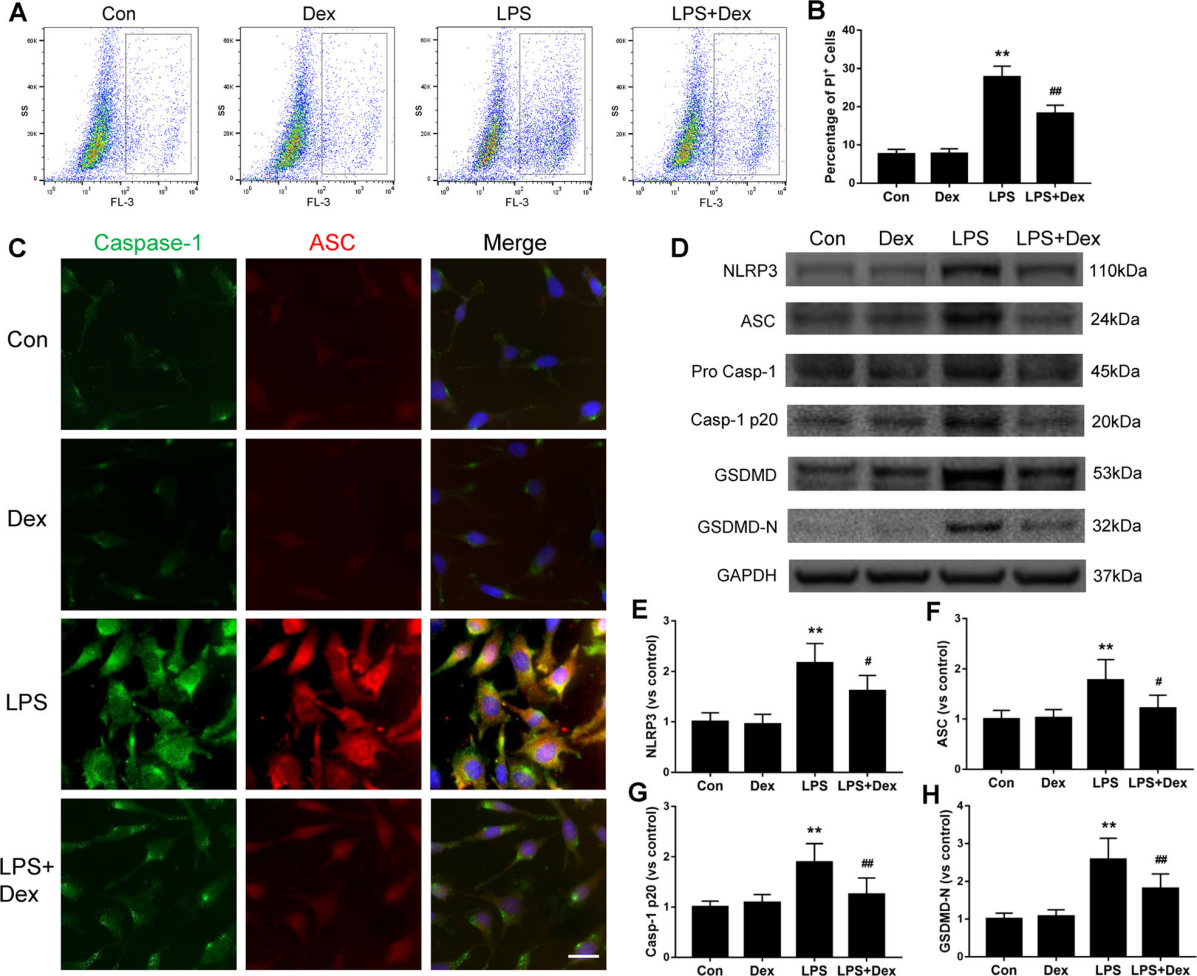
Dexmedetomidine-mediated cytoprotection against LPS-induced pyroptosis in astrocytes. 1321N1 cells were treated with dexmedetomidine (1 µM) for 30 min before supplemented with LPS (100 ng/ml). **a**, **b** Percentage of 1321N1 cell death, measured by propidium iodide (PI) staining followed by flow cytometry analysis. **c** Double immunofluorescent labeling with caspase-1 (green) and ASC (red). Nuclei were counterstained with DAPI. **d**–**h** Expressions of NLRP3, ASC, caspase-1 precursor, caspase-1-p20, GSDMD, GSDMD-N, determined by western blot. Scale bar:  10 µm. Data are represented as mean ± SD (*n* = 6). **p < 0.01, vs. the control group; #*p* < 0.05, ##*p* < 0.01, vs. the LPS group

Dexmedetomidine was found to be cytoprotective against LPS-induced pyroptosis (Fig. 2). Astrocytes treated with dexmedetomidine demonstrated a significant reduction in the PI-positive cells by 34% (*p* < 0.01, Fig. 2a, b) in comparison with that in the LPS group. The expression of ASC and caspase-1 co-localization was evident in cultured astrocytes after exposure to LPS. In addition, the redistribution of ASC protein from nuclei to cytoplasm indicated inflammasome activation, while dexmedetomidine treatment resulted in astrocytes returning to a state similar to the naive control group (Fig. 2c). In addition, dexmedetomidine significantly reduced the expression of NLRP3 by 25% (*p* < 0.05, Fig. 2e), ASC by 32% (*p* < 0.05, Fig. 2f), caspase-1 by 34% (*p* < 0.01, Fig. 2g), and GSDMD by 30% (*p* < 0.01, Fig. 2h) relative to the control. Taken together, these findings indicate that dexmedetomidine ameliorates NLRP3 inflammasome recruitment and caspase-1 activation, as well as decreases pyroptosis in LPS-induced astrocyte injury.

### Dexmedetomidine decreases histone release after LPS exposure in cultured astrocytes

**Fig. 3:**
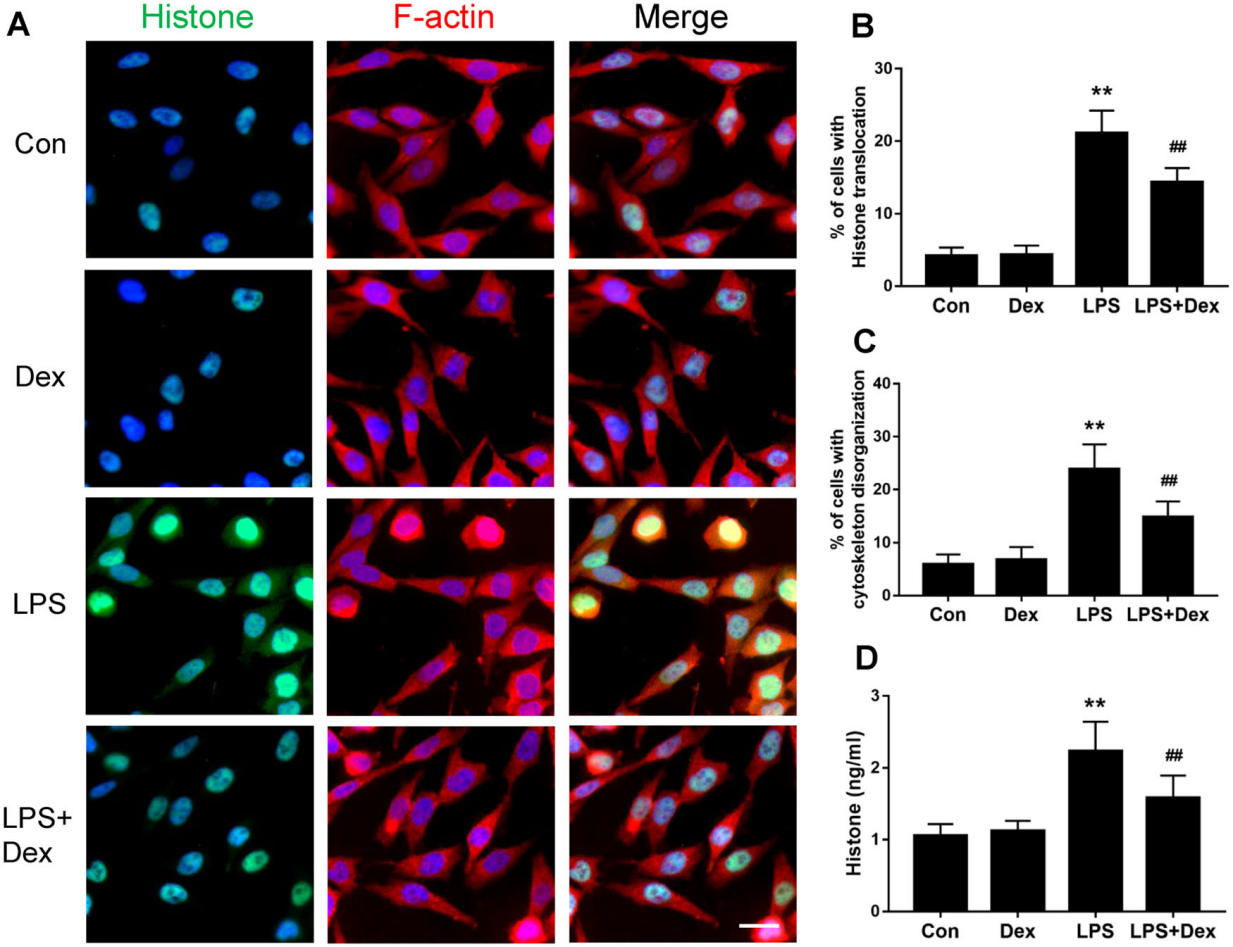
Dexmedetomidine attenuated histone release and cytoskeleton destruction of astrocytes after LPS exposure. 1321N1 cells were treated with dexmedetomidine (1 µM) for 30 min before LPS challenge. **a** Double immunofluorescent labeling with histone H4 (green) and F-actin (red). Nuclei were counterstained with DAPI. Percentage of 1321N1 cells with **b** histone cytoplasm translocation and **c** cytoskeletal disorganization. **d** Concentration of histone H4 in culture medium of treated astrocytes. Scale bar: 10 µm. Data are represented as mean ± SD (*n* = 6). **p < 0.01, vs. the control group; ##*p* < 0.01, vs. the LPS group

Nucleus protein histones can be released to the extracellular space from dying cells during cell injury with enhanced destruction of cellular components. A high level of histone in the cytoplasm was detected after astrocytes were challenged with LPS and demonstrated a more chaotic distribution of cytoskeleton structure in contrast to controls. With dexmedetomidine treatment, histone was restricted, to a large extent, to the nucleus and the cellular integral structure was also preserved (*p* < 0.01, Fig. 3a–c). Quantification of histone in the culture medium of LPS-treated cells revealed a significant increase in the release of histone compared to untreated cells; dexmedetomidine markedly inhibited histone release after LPS challenge (*p* < 0.01, Fig. 3d). These results indicate that dexmedetomidine prevents the disruption of the plasma membrane and maintains cellular morphology.

### Recombinant histone protein induces neurotoxicity in cultured neuronal like cells

**Fig. 4:**
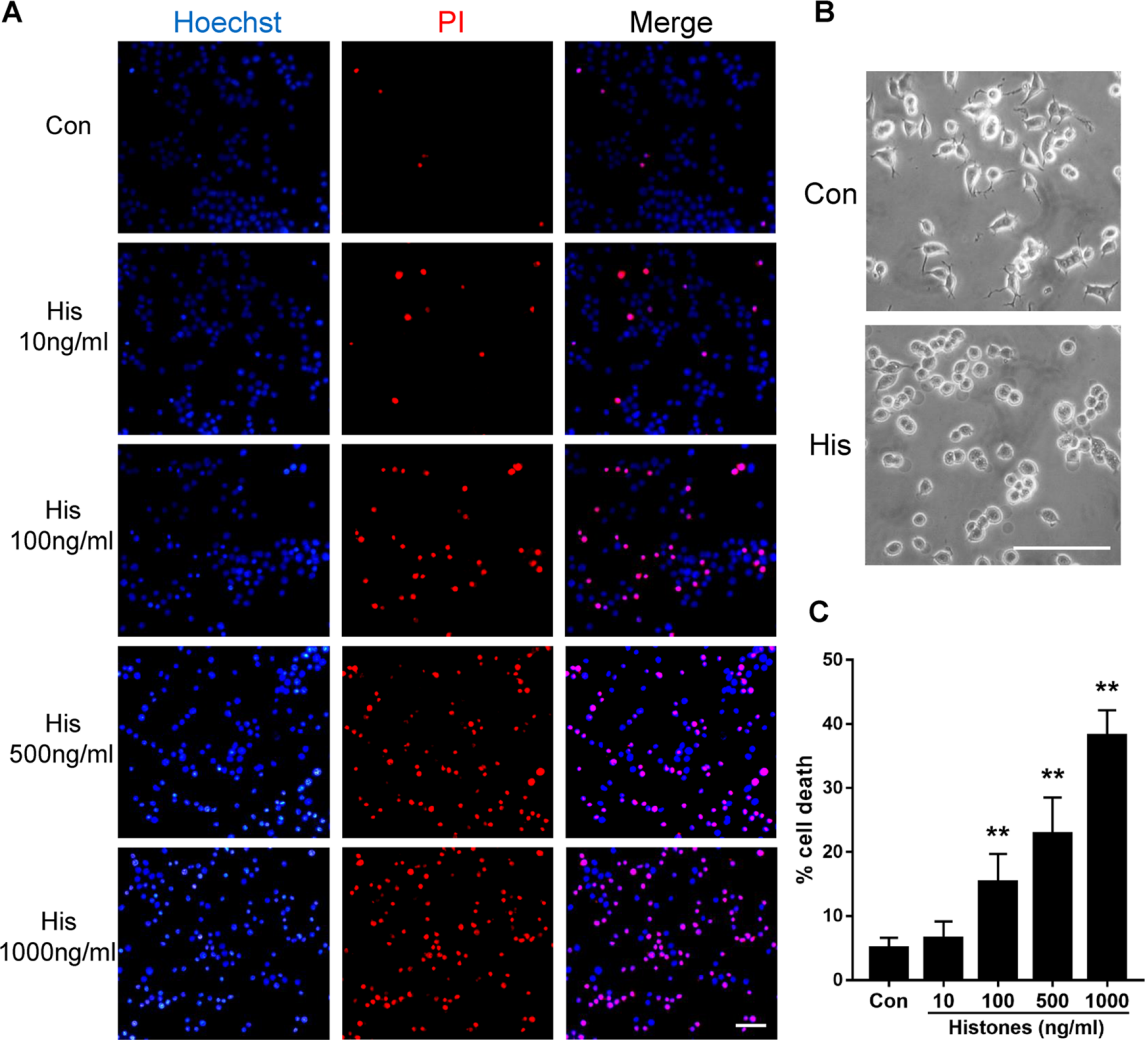
Histone stimuli changed neuron cell morphology and induced toxicity in a dose-dependent manner. Various concentrations of histone recombinant protein were added to PC12 cells for 24 h. **a** Fluorescent microscopy images of Hoechst 33342 and propidium iodide (PI) double staining in cultured PC12 cells showing nuclear morphology and cell death. **b** Morphological appearant changes of PC12 under histones (1000 ng/ml) stimulation. **c** Percentage of dead cells of both Hoechst- and PI-positive cells after normalized to all cells counterstained. Scale bar: 50 µm. Data are represented as mean ± SD (*n* = 6). **p < 0.01, vs. the control group

Extracellular histones are cytotoxic and may damage surrounding milieu during particular types of cell death processes, including pyroptosis. To evaluate the role of endogenously produced histones and their effect on surrounding neurons, we administrated exogenous histones to PC12 cells. As indicated in Fig. 4, toxicity mediated by histones was evaluated by double fluorescent staining with Hoechst 33342 and PI. The number of live cells (Hoechst 33342 normal/PI negative), apoptotic cells (Hoechst 33342 positive/PI negative) and necrotic cells (PI positive) was determined (Fig. 4a). Hoechst and PI staining showed cell death after histone treatment in a dose-dependent manner (*p* < 0.01, Fig. 4c). The morphological pattern of PC12 cells was significantly changed after histone stimulation (Fig. 4b). The cells exhibited small and round cell bodies and short arborizations in comparison with that in the control group, which displayed long distal arborization. Histones, thus, are able to cause neuronal like cell damage following both low-dose and high-dose histone administration.

### Dexmedetomidine mediates neuroprotection against astrocyte pyroptosis and neuroinflammation in septic rats

**Fig. 5:**
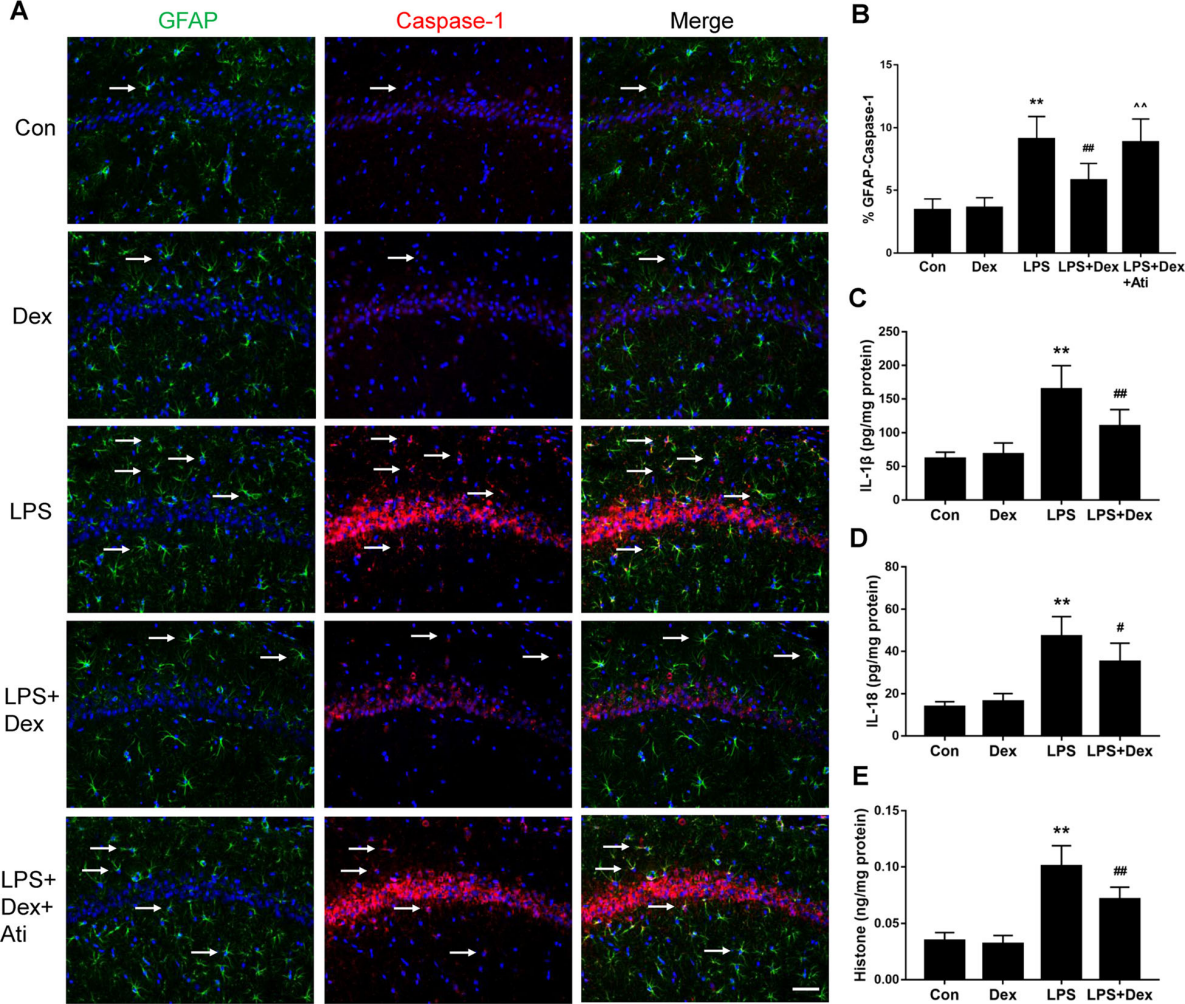
Dexmedetomidine decreased caspase-1 activity of astrocytes and pyroptosis-related inflammation response in septic rats. Brain samples were collected 24 h after LPS administration in the absence or presence of dexmedetomidine or combination with α_2_-adrenoceptor antagonist, atipamezole. **a**, **b** Double immunofluorescent labeling with Glial fibrillary acidic protein (GFAP, green) and caspase-1 (red) in the hippocampus. Nuclei were counterstained with DAPI. **c**–**e** Concentrations of IL-1β, IL-18, histone H4, measured by ELISA. Scale bar: 50 µm. Data are represented as mean ± SD (*n* = 6). **p < 0.01, vs. the control group; #*p* < 0.05 and ##*p* < 0.01, vs. the LPS group; ^^*p* < 0.01, vs. the LPS + Dex group

**Fig. 6:**
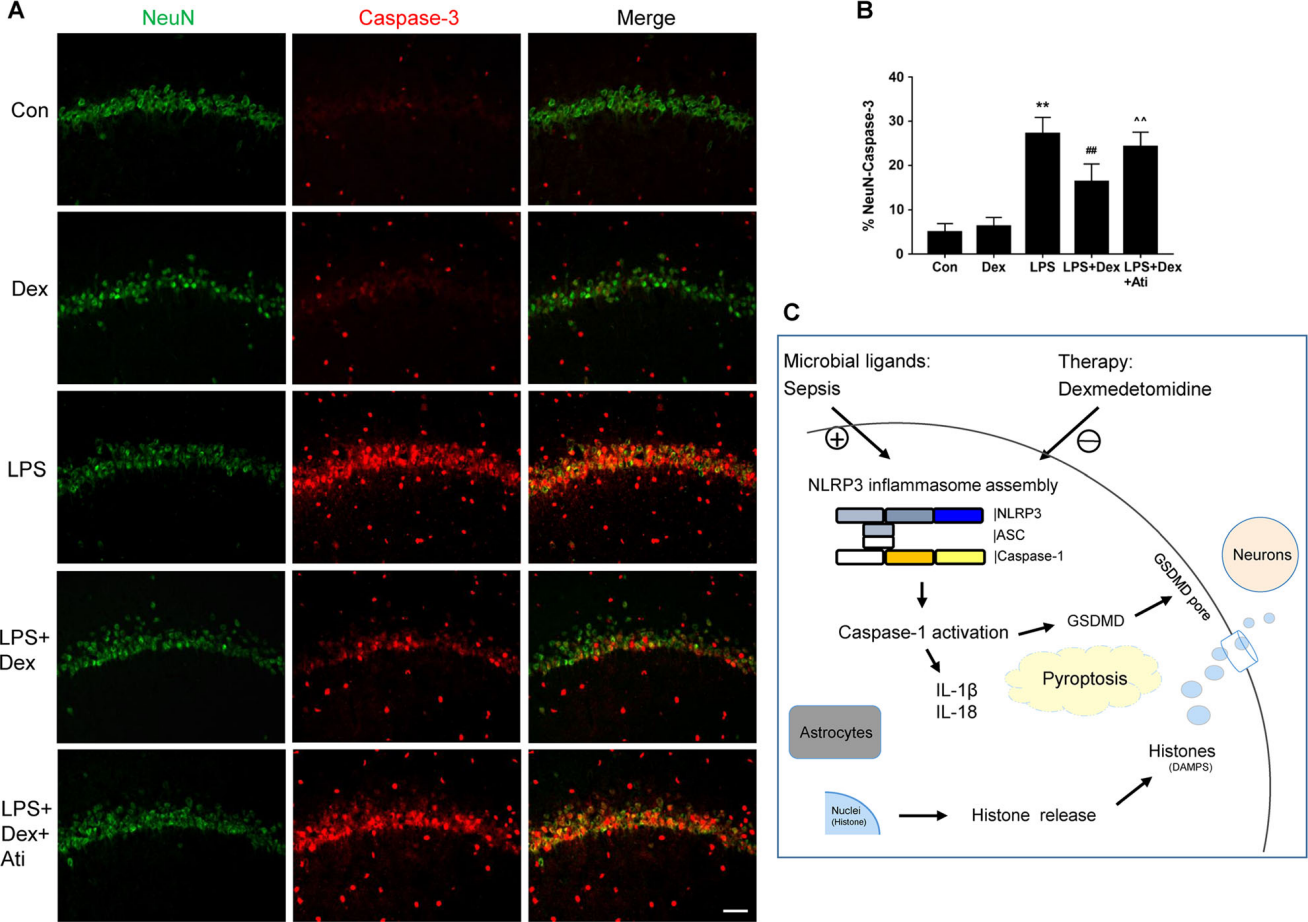
Dexmedetomidine reduced neuron injury in septic rats. Brain samples were collected 24 h after LPS administration with or without dexmedetomidine or combination with α_2_-adrenoceptor antagonist, atipamezole. **a**, **b** Double immunofluorescent labeling with neuronal nuclei (NeuN, green) and caspase-3 (red) in rat hippocampus. Nuclei were counterstained with DAPI. Scale bar: 50 µm. Data are represented as mean ± SD (*n* = 6). **p < 0.01, vs. the control group; ##*p* < 0.01, vs. the LPS group; ^^*p* < 0.01, vs. the LPS + Dex group. **c** Proposed mechanisms of dexmedetomidine in inhibition of pyroptosis in sepsis-induced brain injury. Dexmedetomidine inhibits astrocyte pyroptosis during sepsis. Meanwhile pyroptosis-related inflammation and toxic molecular are suppressed by dexmedetomidine during the cell death process. Dexmedetomidine further protects against neuron injury by attenuating astrocyte pyroptosis

To further confirm our in vitro findings, the effects of dexmedetomidine on brain protection were also examined in vivo. Double immunofluorescent staining of caspase-1/GFAP was performed. LPS treatment increased the expression of caspase-1 activation and resulted in the co-localization of caspase-1 with GFAP-positive cells in the hippocampus of septic rats, when compared with the untreated animals. In contrast, caspase-1 activation was weakly expressed in astrocytes in the dexmedetomidine group compared with that in the LPS group (5.8 ± 1.3% vs. 9.1 ± 1.8%, *p* < 0.01, Fig. 5a, b). The α_2_-adrenoceptor antagonist, atipamezole, blocked the effect of dexmedetomidine-induced caspase-1/GFAP reducing expression (8.9 ± 1.8% vs. 5.8 ± 1.3%, *p* < 0.01, Fig. 5a, b). IL-1β, IL-18, and histone secretion were also determined in the hippocampus of the rats, which were dramatically elevated in the LPS group, compared to the control group. Dexmedetomidine caused a significant reduction in the production of pyroptosis-related cytokines, IL-1β (110.19 ± 24.12 vs. 164.38 ± 35.32, *p* < 0.01, Fig. 5c) and IL-18 (35.25 ± 8.67 vs. 47.22 ± 9.26, *p* < 0.05, Fig. 5d). The reduction in the concentration of histone suggested an attenuation of histone release after dexmedetomidine administration (0.07 ± 0.01 vs. 0.1 ± 0.02, *p* < 0.01, Fig. 5e). Hippocampal neuronal cell injury was determined by caspase-3/NeuN immunostaining in tissue sections. Compared to the control group, an increased accumulation of caspase-3 and an increase in the number of caspase-3-positive neuronal cells (NeuN-positive cells) were also observed in the LPS-challenged rats. The neuroprotective effect of dexmedetomidine was found by suppressing caspase-3/NeuN expression (16.4 ± 4.0% vs. 27.2 ± 3.7%, *p* < 0.01, Fig. 6a, b) whilst atipamezole abolished the caspase-3/NeuN expression downregulation mediated by dexmedetomidine (24.3 ± 3.2% vs. 16.4 ± 4.0%, *p* < 0.01, Fig. 6a, b). Collectively, these findings indicate that dexmedetomidine treatment results in a decrease in neuroinflammation, pyroptosis-associated neuron damage, and histone release in a sepsis model of brain injury (Fig. 6c).

### Dexmedetomidine improves survival in septic rats

**Fig. 7:**
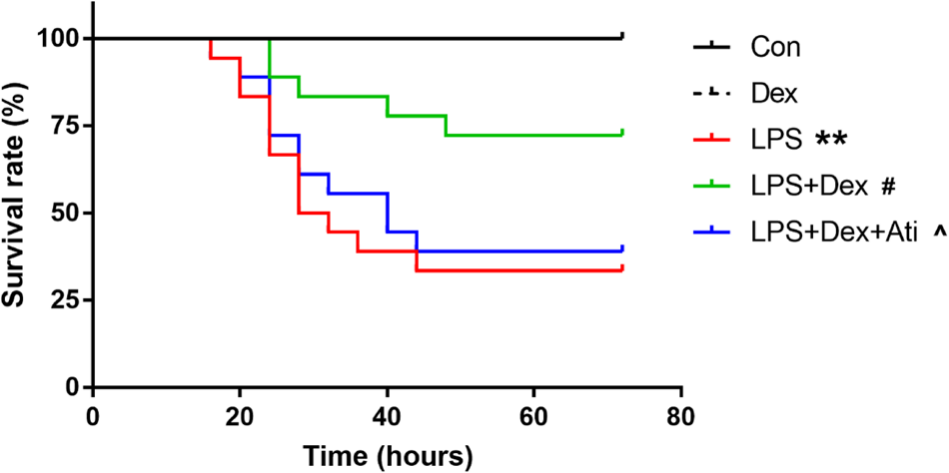
Dexmedetomidine improved survival rate in septic rats. Survival rate within 72 h was analyzed after LPS administration by Kaplan–Meier log-rank test (*n* = 12–18). **p < 0.01, vs. the control group; #*p* < 0.05, vs. the LPS group; ^*p* < 0.05, vs. the LPS + Dex group

Rats exhibited severe sepsis behavior and had a high mortality with LPS injection during the experimental duration of 72 h. Dexmedetomidine significantly increased the survival rate when compared to that in the LPS-treated group (72.22% vs. 33.33%, *p* = 0.0125, Fig. 7). Atipamezole decreased the protective effect of dexmedetomidine (38.89% vs. 72.22%, *p* = 0.0362, Fig. 7).

## Discussion

Studies have demonstrated that dexmedetomidine exhibits neuroprotective effects in various disease models^22–24^; however, the neuroprotective role of dexmedetomidine against a novel form of cell death, pyroptosis, in sepsis-induced brain injury is still unknown. In the present study, we demonstrated that dexmedetomidine exerted brain protection in a model of sepsis in vitro and in vivo. Dexmedetomidine protected brain/neurons by ameliorating astrocyte pyroptosis and harmful neuroinflammation, and improved survival following LPS challenge. Its neuroprotective effect was abolished by α_2_-adrenoceptor antagonist, indicating that the dexmedetomidine provided neuroprotection likely via α_2_-adrenoceptor activation.

During sepsis, innate immune cells are activated by foreign molecular products known as pathogen-associated molecular patterns (PAMPs) molecules, resulting in the activation of inflammatory cells and an increase in pro-inflammatory cytokines^25^. Furthermore, various forms of cell death are involved in sepsis^26–29^. In this study, we chose LPS and TNF-α as a typical example of an endotoxin produced by Gram-negative bacteria, and a main mediator of pro-inflammatory cytokines release during sepsis, respectively. We found that both were able to cause astrocyte death. However, inflammasome activation and GSDMD cleavage were observed in the LPS group, whilst neither of them was appeared in response to TNF-α, indicating LPS but not TNF-α stimuli induced astrocyte pyroptosis during the cell death process.

Pyroptosis, an inflammatory form of programmed cell death, is stimulated by a broad range of pathogens. Driven by inflammasome activation, pyroptosis may contribute to the development of sepsis and septic shock^30^. During pyroptosis, the inflammasome complex is activated and assembled, facilitating caspase-1 activation^14,31^. Subsequently, the inflammatory caspase promotes the cleavage of gasdermin D (GSDMD) protein, which induces pore formation in the plasma membrane, resulting in pyroptosis-induced lytic cell death^32,33^. Among the inflammasome complexes, the NLRP3 inflammasome is the most frequently characterized and has demonstrated a crucial role within CNS. The NLRP3 inflammasome complex is formed following the recruitment of ASC and pro-caspase-1, resulting in inflammasome activation and the initiation of pyroptosis^30^. In addition, pyroptosis has been implicated in the pathogenesis of several neurological diseases, including Alzheimer’s disease, traumatic brain injury, and epilepsy^17,18^.

In this study, the NLRP3 inflammasome and caspase-1 activation were increased in astrocyte cultures treated with LPS. Following NLRP3 inflammasome assembly and activation of its downstream signaling pathways, we observed swollen astrocytes and a significant increase in the cleavage of GSDMD following LPS treatment, thus confirming activation of the pyroptotic death pathway. One of the features of pyroptosis is pore formation in the cell membrane, which can be induced by GSDMD cleavage^32,33^, resulting in rupture of the plasma membrane and release of cytosolic cellular contents including intranuclear proteins^34,35^. Histones are intranuclear proteins that provide structural stability to nuclear chromatin and regulate gene transcription. Furthermore, histones may be released into the extracellular space from dying cells, particularly after necrosis including necroptosis, pyroptosis, and ferroptosis^35^, and act as damage-associated molecular patterns (DAMPs) molecules resulting in further cytotoxicity to surrounding cells. This process has been implicated in sepsis, organ injury and neurodegenerative disease^36,37^. It has been demonstrated the toxic effects of extracellular histone release, which result in endothelial dysfunction, organ failure, and death during sepsis^37^. LPS-induced acute kidney injury in mice partially involves extracellular histones, as indicated by the observation that administration of histone-neutralizing antibodies prevents kidney dysfunction^38^. Exogenous histone administration increases brain infarct volume, whilst anti-H2A/H4 antibody decreases the injury, indicating the possible detrimental effects of extracellular histone in ischemic stroke^39^. Our data demonstrated that histone relocation and release were detectable after LPS treatment, whilst dexmedetomidine restricted histone within the nuclei and decreased histone in the extracellular space. Furthermore, exogenic histones caused neuronal loss, thus indicating that dexmedetomidine further indirectly protected neurons from the cytotoxicity associated with extracellular histones. These data suggest that the release of histones may be exacerbated in LPS-induced pyroptosis, consistent with previous findings which indicate that histones are present in the surrounding extracellular milieu after some forms of cell death, including pyroptosis^35^. Dexmedetomidine may exert its neuroprotective effects by decreasing cytotoxic extracellular histone release during this process.

In this study, we found that dexmedetomidine increased cell survival, and suppressed inflammasome activation and GSDMD augmentation in LPS-induced pyroptotic cell death in astrocytes, indicating that dexmedetomidine was involved in ameliorating astrocyte pyroptosis. This is in line with a recent finding that dexmedetomidine attenuates subarachnoid hemorrhage-associated brain injury by inhibiting the NLRP3 inflammasome pathway^40^.

Although dexmedetomidine protected neurons has been well demonstrated in various models of brain injuries^22,24,41,42^, its protective effect on astrocytes has not been reported yet. Astrocytes are an important immunological component of the central nervous system (CNS), participating in synaptic plasticity and information processing in the neuronal circuit. They exert metabolic effects and provide structural support for neurons, and also play a role in modulating neuron homeostasis and higher neuronal functions. The interaction between astrocytes and neurons plays a crucial role in the development and progression of diverse neurological disorders. Alterations in the physiological function of astrocyte induce the loss of essential neurosupportive and neuroprotective functions, resulting in several cerebral disorders^43^. Microglia, as the second most abundant glia cell types in the CNS^44^, are activated quickly under pathologic stimuli, e.g. LPS, which, in turn, release various pro-inflammatory mediators within the CNS, leading to highly “inflammable” inflammatory state. In addition, microglia are activated earlier than astrocytes and facilitate astrocyte activation. Subsequently, activated astrocytes in turn modulate activities of microglia and also promote activation of distant microglia^45^. They eventually “collaborate” with each other and enhance inflammatory responses in the pathological conditions such as sepsis-caused neuroinflammation and finally result in neuronal injury and brain functional impairment. Moreover, previous studies demonstrated that over-activated astrocytes themselves could eventually undergo cell death in presence of different insults^6,46^, and those astrocytes were unable to provide their homeostatic function and protect neurons; all of which contributed to the development of the deleterious neurological sequelae^6,47^. Therefore, as the first line of CNS defense, it is important to protect astrocytes from pathological insults in order to further protect neurocentric brain function. Furthermore, peripheral LPS administration may trigger inflammasome activation and the production of inflammatory mediators within the brain^48^. Studies have demonstrated that the development of neuroinflammation-related brain disorders is associated with a significant increase in caspase-1^17^. Caspase-1, a core feature of pyroptosis, is activated during pyroptosis, initiating the release of mature IL-1β and IL-18. Prolonged elevation of IL-1β and IL-18 causes neurotoxicity^49,50^; and high concentrations of IL-1β and IL-18 have been demonstrated in the cerebrospinal fluid (CSF) and brain tissue of patients with CNS infection, brain injury, and neurodegenerative disorders^51–53^. In this study, we observed an elevated level of caspase-1 immunoreactivity in astrocyte cells, as well as an increase in IL-1β and IL-18 release and histone production in the brain of septic rats. Furthermore, neuronal injury was observed following LPS administration, indicating that pyroptosis may play a vital role in the development of neuroinflammation and sepsis-induced brain injury, whilst impaired astrocytes failed to effectively provide neuronal support. Dexmedetomidine abolished the above detrimental changes and protected both glia and neurons and ultimately preserved brain functions *per se*. Indeed, it reduced coma free time when used in ICU for septic patients^54^.

It is worth mentioning that except attenuating inflammation and protecting glia cells and neurons afforded by dexmedetomidine as discussed above, its organ protections in particular on brain, kidney, and lung were well documented previously^12,22,42,55–60^. In addition, dexmedetomidine can promote natural sleep whilst natural sleep is important in maintaining physiological homeostasis including preserving immunofunction and restoring body energy and repairing potential inherent injury^61–64^. Owning to all above but not limited favorable effects of dexmedetomidine, it effectively decreased the mortality induced by LPS found in the current study which was in line with our previous report^65^.

There are indeed some limitations. Although the dexmedetomidine concentrations used in our experiment have been repeatedly reported previously^22,57,60,66^, it cannot be denied that there is still a gap between laboratory study and clinical practice. It has been shown that the therapeutic concentration of dexmedetomidine in the human plasma can be reached up to 1 µM after intravenous infusion^67^. It was also reported that 25 µg/kg dexmedetomidine provided significant neuroprotective effect against isoflurane-induced injury in the young^22^. Those work may arguably provide a basis of the doses of dexmedetomidine that were chosen for our in vitro and in vivo study, respectively. However, it must be admitted that the dose of dexmedetomidine is considerably higher than the dose used clinically. Once can appreciate is that drug used in animals in general is normally 7–10 times higher than used in humans.

Collectively, to our acknowledge, this study demonstrated, for the first time, that dexmedetomidine protected against pyroptosis in sepsis-induced brain injury. These data suggest that dexmedetomidine may enhance neuronal survival by inhibiting astrocyte pyroptosis and, therefore, decrease potentially harmful pyroptosis-related inflammatory responses within brain, indicating a novel neuroprotective mechanism of dexmedetomidine in sepsis-induced brain injury.

## Materials and methods

### Cell lines and cell treatments

Human astrocyte 1321N1 cells and rat neuron PC12 cells were purchased from the European Collection of Authenticated Cell Cultures (ECACC, Porton Down, UK). 1321N1 cells were cultured in Dulbecco’s modified Eagle’s Medium (DMEM) (Gibco; Invitrogen, USA) supplemented with 10% fetal bovine serum and 100 U/ml penicillin-streptomycin. Some cohort 1321N1 cultures were treated with lipopolysaccharide (LPS) *E. coli* O111:B4 (100 ng/ml, Sigma-Aldrich, Poole, UK) for 24 h, some cohort cultures were treated with 1 µM dexmedetomidine (Sigma-Aldrich) 30 min before LPS treatment^22^, and some cohort cultures were treated with TNF-α (100 ng/ml, Sigma-Aldrich) for 24 h. PC12 cells were maintained in RPMI-1640 medium (Gibco; Invitrogen, USA) containing 10% heat-inactivated horse serum, 5% fetal bovine serum and 100 U/ml penicillin-streptomycin. To induce the differentiation of PC12 cells into neuronal like cells, 50 ng/ml nerve growth factor (NGF) was added into the medium with 1% horse serum and cells were seeded on a Collagen IV coated dish for 24 h. The medium was routinely replaced with fresh medium every 2 days for 5 days until neuronal differentiation had taken place. PC12 cells were treated with calf thymus histones (1000 ng/ml, Sigma-Aldrich) for 24 h. The cells were incubated at 37 °C in a humidified atmosphere with 5% CO_2_.

### Animals and drug administration

The experimental procedures were approved by the Peking University Biomedical Ethics Committee experimental animal ethics branch (Approval No. J201807) and performed in accordance with the institutional guidelines. Adult male Sprague-Dawley rats (250–300 g) were purchased from the Experimental Animal Center of Peking University Health Center (Beijing, China). Rats were housed in standardized conditions with room temperature 24 ± 2 °C, controlled humidity 55 ± 5%, 12 h light/12 h dark cycle and access to water *ad libitum*. Rats were injected intraperitoneally with 10 mg/kg LPS (Sigma-Aldrich), dissolved and diluted in sterile normal saline. Dexmedetomidine (Sigma-Aldrich) was then intraperitoneally injected at a dose of 25 µg/kg every 2 h for three times immediately after LPS administration^22^. One cohort was injected intraperitoneally with 500 µg/kg α_2_-adrenoceptor antagonist atipamezole (Sigma-Aldrich) every 2 h in three doses prior to the administration of dexmedetomidine^22^. Controls received comparable volume injections of saline. At 24 h after the first treatment, animals were sacrificed with pentobarbitone (100 mg/kg, i.p.). Their hippocampus was quickly dissected and snap frozen in liquid nitrogen from the half of the animals from each group. The remaining animals were perfused with 4% paraformaldehyde and their brains were collected.

### Flow cytometry

Propidium iodide (PI; Sigma-Aldrich) staining was used to measure cellular death. Cells were collected in a fluorescence-activated cell sorting (FACS) tube and washed twice before resuspension in FACS buffer. PI was added to make the final concentration to 1 µg/ml and incubated in the dark for 5 min at room temperature. PI fluorescence was detected with flow cytometry. Each assay included at least 10,000 gated events.

### Western blot analysis

The lysates from cultured cells were centrifuged, the supernatant was collected, and total protein concentration was quantified by the Bradford protein assay (Bio-Rad, Hemel Hempstead, UK). The protein extracts (80 µg per sample) underwent SDS-polyacrylamide gel electrophoresis and were then transferred to a polyvinylidene difluoride membrane. The membranes were blocked with 5% non-fat dried milk and probed with primary antibodies: anti-NLRP3 (1:1000, Cell Signaling Technology), anti-ASC (1:500, Santa Cruz), anti-caspase-1 (1:1000, Abcam) and anti-GSDMD (1:1000, Novus Biologicals) in TBS-T overnight at 4 °C, followed by HRP-conjugated secondary antibody for 1 h. The loading control was GAPDH (1:10,000, Millipore). The blots were detected with enhanced chemiluminescence (ECL) system (Santa Cruz Biotechnology) and analyzed with GeneSnap (Syngene, Cambridge, UK). The protein band intensity was normalized with GAPDH and expressed as ratio of the control.

### Immunofluorescent staining

For in vitro fluorescent staining, cells were fixed in 4% paraformaldehyde in 0.1 mol/L PBS solution, then blocked with 10% normal goat serum for 1 h and incubated with the primary antibodies: rabbit anti-caspase-1 (1:200, Abcam), mouse anti-ASC (1:200, Santa Cruz), rabbit anti-histone H4 (1:200, Abcam), mouse anti-F-actin (1:200, Abcam) overnight followed by fluorescently conjugated secondary antibodies for 1 h. For in vivo fluorescent staining, brains were collected and fixed in 4% paraformaldehyde for 16 h at 4 °C, followed by dehydration in 30% sucrose solution for 24 h at 4 °C. Brain samples were then cryosectioned at −20℃ into14 µM slices and mounted onto slides. Sections were rinsed in 0.1% Triton in PBS and incubated with 10% normal goat serum. Sections were then incubated overnight with rabbit anti-caspase-1 (1:200, Abcam), mouse anti-glial fibrillary acidic protein (GFAP) (1:200, Sigma-Aldrich), rabbit anti-caspase-3 (1:200, Cell Signaling Technology), mouse anti-neuronal nuclei (NeuN) (1:200, Millipore), followed by secondary antibodies. For double-labeled immunofluorescence, cells and tissue samples were incubated with the first primary antibody overnight, followed by the first secondary antibody, and then the second primary antibody and the second secondary antibody. The slides were counterstained with nuclear dye 4′,6-diamidino-2-phenylindole (DAPI) and mounted with Vectashield mounting medium (Vector Laboratories, USA). Ten high-power fields at ×20 magnification were photographed using an AxioCam digital camera (Zeiss, Welwyn Garden City, UK) mounted on an Olympus BX60 microscope (Olympus, Middlesex, UK) with Zeiss KS-300 software (Zeiss, Welwyn Garden City, UK).

### Hoechst and PI staining

PC12 cells were co-stained with Hoechst 33342 (1 µg/ml, Sigma-Aldrich) and PI (5 µg/ml, Sigma-Aldrich) for 20 min at room temperature, then stained nuclei were observed under a fluorescent microscope^68^. Cell death was identified on the basis of positive staining with PI and apoptotic nuclear morphology changes with Hoechst dye.

### Enzyme-linked immunosorbent assay

The cell medium histone H4, brain tissue IL-1β, IL-18 (Neobioscience Technology, Shenzhen, China) and histone H4 (USCN Life Science, Wuhan, China) concentrations were measured by using enzyme-linked immunosorbent assay (ELISA) kits following the manufacturer’s instructions.

### Survival analysis

Rats receiving the same interventions as indicated were maintained under normal housing conditions. They were monitored every 4 h and killed once they reached humane endpoints (significant body weight loss, immobilized, very poor grooming, and back arching). The survival curve was constructed.

### Statistical analysis

The statistical analyses were performed with SPSS version 20.0. Data were expressed as mean ± SD and analyzed by one-way analysis of variance (ANOVA) with Bonferroni’s post hoc test or Student’s *t*-test. A *p*-value < 0.05 was considered to be statistically significant. Animal survival analysis was performed using Kaplan–Meier survival estimates, and statistical significance was analyzed by the log-rank test (GraphPad Prism 5.0; GraphPad Software).
